# Varicella zoster virus meningoencephalitis and vasculopathy in an immunocompromised patient with rheumatoid arthritis: a diagnostic challenge

**DOI:** 10.1007/s13365-026-01306-w

**Published:** 2026-02-02

**Authors:** Maria Lima, Mantatzis Michail, Ioannidis Panagiotis, Grigoriadis Nikolaos, Afrantou Theodora

**Affiliations:** 1 B’ Department of Neurology, Aristotle University of Thessaloniki, AHEPA University General Hospital of Thessaloniki, St. Kiriakidi Street 1, Thessaloniki, 54636 Greece; 2 Radiology Department, Aristotle University of Thessaloniki, AHEPA University General Hospital of Thessaloniki, St. Kiriakidi Street 1, Thessaloniki, 54636 Greece

**Keywords:** Varicella Zoster Virus (VZV), Rheumatoid Arthritis, Meningoencephalitis, Vasculopathy, Antibody Index

## Abstract

The purpose of this paper is to describe a challenging case of a Varicella Zoster Virus (VZV) meningoencephalitis-vasculopathy in an immunocompromised patient with severe Rheumatoid Arthritis (RA), emphasizing on diagnostic tools. Hereby, we present an elderly immunocompromised patient with RA that developed meningoencephalitis and Central Nervous System (CNS) vasculitis, only two months after discontinuation of her RA medication due to a VZV rash. The clinical, imaging and laboratory investigation that was performed led to a diagnostic dilemma, as it could argue in favor both of VZV infection and RA activation in CNS. The intrathecal production of VZV-IgG antibodies and “Antibody Index” are fundamental tools in the diagnosis of VZV vasculopathy, rather than the presence of virus in CNS itself. Based on this finding in our patient and taking into consideration that RA vasculopathy is rarer, the patient was treated with acyclovir and methylprednisolone as indicated for VZV meningoencephalitis-vasculopathy. Since the therapeutic approach of CNS complications in immunocompromised patients is an additional challenge, the correct diagnosis becomes even more necessary. This article highlights the diagnostic tools that can assist neurologists making the correct diagnosis promptly, even when biopsy is not possible or undesirable.

KEYWORDS:

## Introduction

Varicella Zoster Virus (VZV) is the Human Herpesvirus type 3 that can cause varicella when it first infects humans. More than 90% of world population has established latency of VZV, due its ability to resident in peripheral autonomic and sensory ganglia in the dorsal roots and cranial nerves, that in more than half of them would be reactivated by 85 years old, leading to Herpes Zoster (shingles) (Nagel et al. 2020). Patients with autoimmune diseases, like Rheumatoid Arthritis (RA), or others that are under immunomodulatory treatment, are in greater risk of reactivation of VZV (Kennedy and Mogensen 2020). Especially immunocompromised elderly are in greater risk for VZV Central Nervous System (CNS) involvement, like meningoencephalitis or VZV-Vasculopathy (Gilden et al. 2000).

We hereby present a difficult case of an immunocompromised RA patient with VZV Meningoencephalitis and Vasculopathy, elucidating the difficult diagnostic approach of the cause of this complication between RA itself and VZV infection.

## Case presentation

An elderly woman was admitted to emergency department in a comatose state, Glascow Coma Scale (GCS): 1-1-5 (7/15), due to bilateral convulsive status epilepticus (SE) of focal onset of the right upper limb. The patient received diazepam 10 mg (iv), midazolam 10 mg (iv), phenytoin 1gr (iv) as an emergency treatment of SE. From her medical history, the patient suffered from severe RA with almost daily fevers. She was under prednisolone 2.5 mg, leflunomide 20 mg, baricitinib 4 mg, and hydroxychloroquine 200 mg, which were discontinued about two months ago due to a VZV rash on the right hemithorax, for which she had completed treatment with acyclovir 800 mg five times daily for one week and now receiving pregabalin 75 mg twice daily for postherpetic neuralgia.

**Fig. 1:**
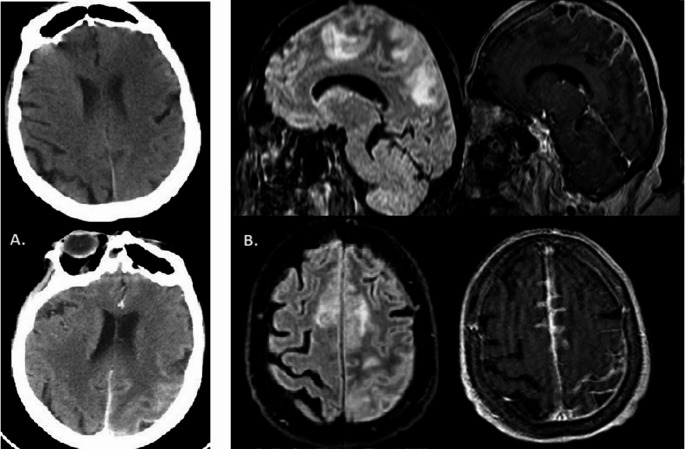
**(A)** Brain CT pre and post contrast. A hypodense area at the left corona radiata parietally. There is a slight enhancement following the cortex. (**B) **Brain MRI, FLAIR and T1-WI post contrast sequences, parasagittal and axial planes: There is increased signal intensity in the subcortical white matter in the left parietal lobe and superior frontal gyrus bilaterally. The cortex looks slightly swollen in the left superior parietal lobule.Enhancement within sulci of the left parietal and in a smaller extent to the interior surface of superior frontal gyrus

**Fig. 2:**
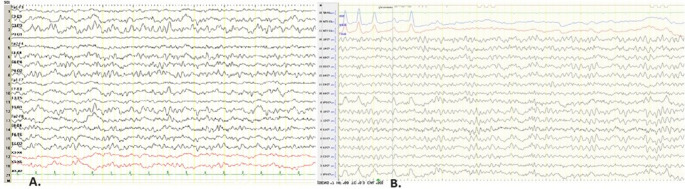
**(A)** Initial EEG: Bipolar montage. Μild generalized slowing, and continuous recording of slow multiform abnormalities 2.5-4 Hz in the temporoparietal regions of the left hemisphere with extension to the homologous regions of the opposite hemisphere. **(B)** Follow-up EEG: Reference montage (Cz). (LEFT). Recording of single slow multiform waves freq. 2 – 4 Hz in the left posterior temporal-parietal region. Improvement of findings compared to previous EEG. (Machine settings display: sensitivity 10μv/mm, time constant (s) o.3s, high frequency filter 70Hz, paper speed 10 sec per page/screen)

Her neurological examination revealed a right hemiparesis (muscle strength 2/5) which improved within 48 h and was attributed to Todd’s palsy. An emergency brain Computerized Tomography (CT) showed a hypodense area at the left corona radiata extending to posterior limb of the internal capsule (Fig. 1.A) and electroencephalogram (EEG) in the next 24 h revealed a mild generalized slowing, and continuous recording of slow multiform abnormalities 2.5–4 Hz in the left temporoparietal region (Fig. 2.A). The patient was treated with phenytoin 125 mg x3 (iv) and acetylsalicylic acid 100 mg. From rheumatological screening, there were high levels of C-Reactive Protein (CRP) [20 − 10 mg/dl; normal values (nv) < 0,5], Erythrocyte Sedimentation Rate (ESR) (103–140 mm; nv: 0–20 mm), Rheumatoid Factor (RF) (24–61 IU/ml; nv: 0–20 IU/ml) and anti – Cyclic Citrullinated Peptide (anti-CCP) (> 200 RU/ml; nv < 5 RU/ml). High anti-CCP antibodies is a specific diagnostic and prognostic indicator related to the extra-articular manifestations and activity of RA. The patient’s chest CT showed three rheumatoid nodules up to 10 mm subpleurally with reactive axillary lymph nodes.

**Fig. 3:**
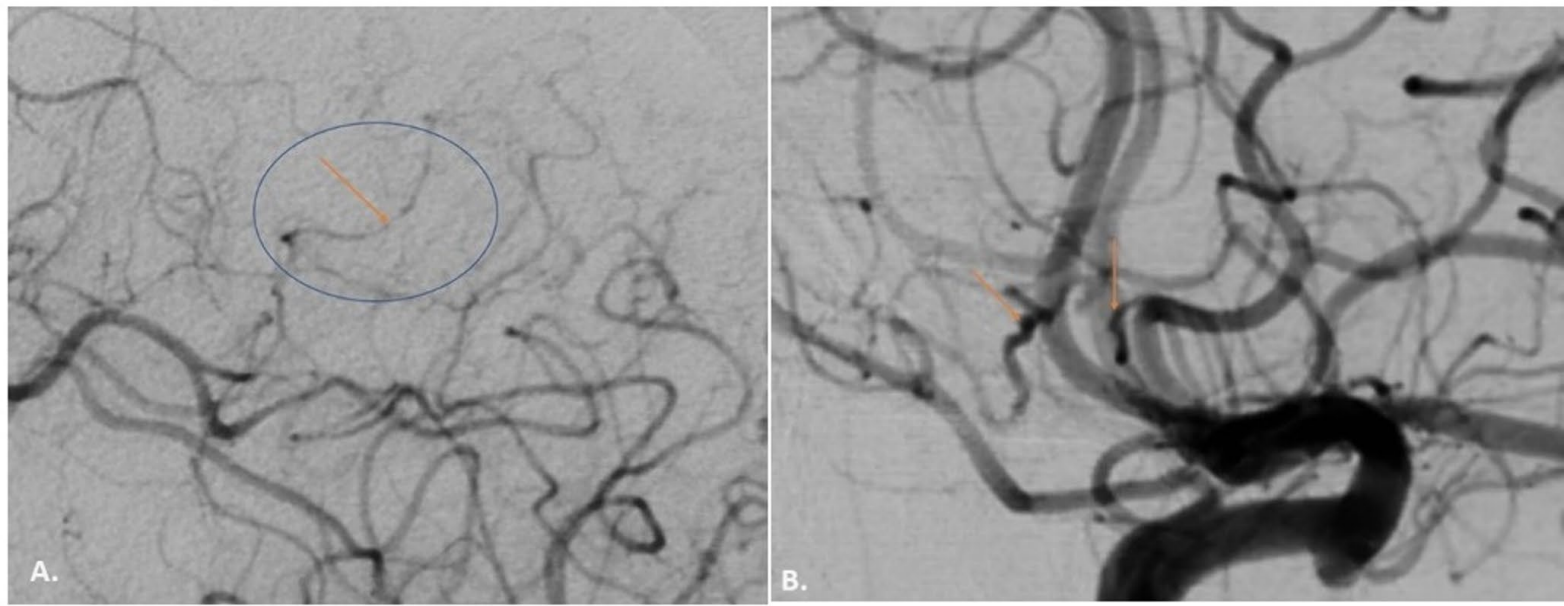
Brain– DSA.** (A)** Significant segmental narrowing of peripheral branches (A4-A5) of the anterior cerebral artery. **(B)** Selective injection of the left MCA. Sequential aneurysmal dilation in peripheral branches

During her hospitalization the patient had a gradual worsening of her condition with right spastic-ataxic hemiparesis progressing to spastic tetraparesis with positive Babinski sign bilaterally, dizziness, slurred speech, confusion and recurrence of focal motor seizures of the right lower limb. Lumbar puncture was performed, which showed 7–8 mononuclear cells (lymphocytes and monocytes), 89.9 mg/dl total protein (nv < 45) and Oligoclonal Bands type 3. Phenytoin therapy was modified to the upper limits of plasma drug levels (20–25 µg/ml) with the addition of levetiracetam 500 mg twice daily, while she did not tolerate clonazepam, oxcarbazepine, and gabapentin due to severe dizziness. The Brain MRI (Fig. 1.B) showed increased signal intensity on T2-weighted and FLAIR sequences, involving the cortex and its subcortical white matter in a large area in the left parietal lobe and smaller areas of the superior frontal gyrus bilaterally with concomitant diffusion restriction and gadolinium enhancement within brain sulci in the corresponding regions. The findings were attributed mainly to leptomeningitis of inflammatory etiology, with an underlying reactive swelling of the parenchyma and suspicion of vasculitis. A Brain Digital Subtraction Angiography (DSA) was then performed, which was indeed indicative for vasculitis (Fig. 3). Meanwhile, a comprehensive virological screening in serum and CSF argued in favor of VZV infection of the CNS:

- serum IgG-VZV antibodies (> 150 AU/ml; nv < 8 AU/ml).
- serum IgM-VZV antibodies (100 AU/ml, nv < 8AU/ml).
- CSF IgG-VZV antibodies (32,9 AU/ml; nv < 8AU/ml) – without IgM antibodies.
- VZV–DNA (1400 copies/ml) in CSF.

To better confirm the *intrathecal* production of VZV-IgG antibodies, we calculated the Antibody Index (AI) = Qspec/QIgG (Kronenberg et al. 2002; Reiber and Lange 1991).

A $$AI=IgG-VZV\;serum/IgG-VZV\;CSF=5$$

B $$Total\;IgG\;ratio=Total\;IgG\;in\;serum/Total\;IgG\;in\;CSF=49$$

C $$Albumin\;ratio=Albumin\;serum/Albumin\;CSF=11,6$$

First of all, (A) should be lower than (B) and (C), which applies in our case.

(A) < (B) and (A) < (C).

Secondly, the normal range for AI is **=** 0.7–1.5. To confirm *intrathecal* production of IgG against a specific virus, AI should be abnormal. In our case:

- ΑΙ = Qspec/QIgG = (IgG-VZV CSF/serum) **/** (IGG total CSF/serum) = 11.

- AI = 11 > 1,5.

That means that VZV-IgG is produced *intrathecal* and CNS vasculitis was also attributed to VZV-Vasculopathy.

Consequently, the patient received as indicated, a 14-day intravenous regimen of acyclovir 750 mg (10–15 mg/kg) every 8 h and a 4-day regimen of intravenous methylprednisolone 500 mg with oral tapering. Soon after the first days of treatment a significant improvement in her neurological condition was noted, which was also reflected in the EEG (Fig. 2.B). Our patient was discharged from the hospital continuing with oral valacyclovir 1gr daily, as it is indicated in susceptible immunocompromised patients, and was referred to her attending rheumatologist.

## Discussion

The above case demonstrates the difficulty of managing immunocompromised patients with complex neurological complications in order to find the exact cause of the problem. *On the one hand*, the patient had a heavy form of RA, discontinued her RA medication and had high indicators of RA activity (especially anti-CCP) indicating extraarticular manifestations. Vasculopathy and Rheumatoid Meningitis (RM) could be a CNS manifestation of RA (Trabelsi et al. 2020; Watts et al. 2004). RM is an aseptic inflammation of meninges, described in MRI as pachy- and/or lepto-meningeal thickening with contrast enhancement, not firmly specific of RM (Trabelsi et al. 2020). RA Vasculopathy is also a rare and diagnostically challenging disorder as few cases have been reported in patients with RA (Jayaraman et al. 2018).

*On the other hand*, considering that our patient had months ago a herpetic rash, it would be reasonable to assume that reactivation of VZV could be responsible for her condition. Patients with VZV-Vasculopathy have a rash in around four months before, which, however, may be absent in 43% of cases. Imaging findings for vasculitis should be present but are not specific. In half of cases there is CSF pleocytosis (up to 67%), commonly monocytes. Last but not least, intrathecal production of anti-VZV IgG in CSF has high sensitivity and specificity in diagnosis of VZV-Vasculopathy (Lyman et al. 2023), which was also confirmed with AI. PCR testing of CSF is estimated at 30% sensitive for VZV – DNA; its absence does not exclude the diagnosis (Nagel et al. 2007, 2020; Kennedy and Mogensen 2020; Gilden et al. 2000). Our patient had all of the above-mentioned findings, treated with the proper treatment and finally improved. A minor caveat of this report is the absence of follow-up information from the treating rheumatologist, regarding the subsequent course of her disease-modifying therapy, though this did not affect the neurological assessment presented here.

To confirm the exact cause, when the laboratory data do not converge in favor of a diagnosis, a brain biopsy is required, and this could be also considered a limitation of our case report. However, a brain biopsy is often high-risk in these patients, and a specialized laboratory is also required for a safe histological diagnosis. For this reason, it is very important to highlight the importance of CSF-serum findings, since imaging findings are not specific, and the aid of AI of intrathecal production of VZV-IgG antibodies in the differential diagnosis of these conditions.

## Data Availability

No datasets were generated or analysed during the current study.
