# Correction: Varicella zoster virus meningoencephalitis and vasculopathy in an immunocompromised patient with rheumatoid arthritis: a diagnostic challenge

**DOI:** 10.1007/s13365-026-01308-8

**Published:** 2026-02-17

**Authors:** Maria Lima, Michail Mantatzis, Panagiotis Ioannidis, Nikolaos Grigoriadis, Theodora Afrantou

**Affiliations:** 1 B’ Department of Neurology, Aristotle University of Thessaloniki, AHEPA University General Hospital of Thessaloniki, St. Kiriakidi Street 1, Thessaloniki, 54636 Greece; 2 Radiology Department, Aristotle University of Thessaloniki, AHEPA University General Hospital of Thessaloniki, St. Kiriakidi Street 1, Thessaloniki, 54636 Greece

The given names and surnames of some of the authors were inadvertently transposed. The corrected author names are Maria Lima, Michail Mantatzis, Panagiotis Ioannidis, Nikolaos Grigoriadis, and Theodora Afrantou. 

